# Foot and Ankle Conventional Radiography in Juvenile Idiopathic Arthritis: Does It Still Matter?

**DOI:** 10.5334/jbsr.2729

**Published:** 2022-09-23

**Authors:** Magdalena Posadzy, Anna Sowińska, Filip Vanhoenacker, Piotr Gietka, Ewa Żelnio, Iwona Sudoł-Szopińska

**Affiliations:** 1 Indywidualna Praktyka Lekarska Magdalena Posadzy, Poznan, PL; 2 Poznan University of Medical Sciences, PL; 3 AZ Sint-Maarten Mechelen and University (Hospital) Antwerp/Ghent, BE; 4 National Institute of Geriatrics, Rheumatology and Rehabilitation, Warsaw, PL; 5 Department of Radiology, National Institute of Geriatrics, Rheumatology and Rehabilitation, Warsaw, PL

**Keywords:** Foot, Ankle, Radiography, Arthralgia, Juvenile Idiopathic Arthritis (JIA)

## Abstract

**Objectives:**

The aim of this study was to evaluate the residual value of Conventional Radiography in children with arthralgia clinically suspected of Juvenile Idiopathic Arthritis (JIA).

**Materials and Methods:**

Three hundred seventy-two patients aged 1–18 years suspected of JIA were retrospectively reviewed. All patients had foot and ankle plain films performed in standard two projections: ankle in antero-posterior and lateral, and foot in antero-posterior and oblique. The cohort was divided into two groups: patients with confirmed JIA and non-JIA control group of children with foot and ankle arthralgia without diagnosis of inflammatory connective tissue disease. Radiographic findings in both groups were compared.

**Results:**

In 40% of JIA and 70% of non-JIA patients radiographs were normal. All radiographic findings were significantly more common in JIA than in non-JIA group (p = 0.000). Soft tissue swelling was the most frequent abnormality found in JIA patients (31, 51%) and only in 2.41% of non-JIA patients (p = 0.000). Osteoporosis and joint space narrowing were also significantly more common in JIA group (p = 0.000). The majority of imaging findings in non-JIA group were non-inflammatory abnormalities.

**Conclusion:**

Conventional radiography is an important tool in differential diagnosis of arthralgia of unknown etiology, as soft tissue swelling, osteoporosis and joint space narrowing are significantly more common in JIA patients as compared with patients without the diagnosis of inflammatory connective tissue disease. However, in case of high clinical suspicion of JIA and normal radiography, we recommend subsequent ultrasound (US) and/or MRI to allow early treatment.

## Introduction

Foot and ankle involvement in juvenile idiopathic arthritis (JIA), although less frequent than knee joint or wrist arthritis, is considered an important predictor of unfavorable disease outcome [1,2]. Ankle arthritis tends to occur early in the course of the disease and may affect up to approximately 60% of patients [1,3]. Given the potential progression from JIA to the active adult form of rheumatic disease accounting in up to 55% of cases with severe disability occurring even in 5% patients [4], the need for correct and early diagnosis remains indisputable. The diagnosis of JIA is based on clinical assessment, supplemented with laboratory tests; both however are nonspecific [5]. Imaging is essential in the classification, treatment planning, monitoring, prognostication as well as in the detection of arthritis with subclinical manifestation. Radiographs are most valuable in the detection of chronic, destructive lesions [4,6,7] and growth disturbances [4,8].

The purpose of this study was to investigate the radiographic findings in patients with foot and ankle arthralgia clinically suspected of JIA and to discuss whether radiography can be useful to distinguish JIA and non-JIA disorders.

## Materials and Methods

The study group included 372 patients aged 1–18 years hospitalized in pediatric rheumatologic clinic with foot and ankle arthralgia clinically suspected of JIA [9]. Children with history of previous trauma, immobilization, congenital deformities and surgical procedures at the level of foot and ankle were excluded.

In all patients, plain films of the foot and ankle in standard two projections were performed (ankle in antero-posterior [AP] and lateral and foot in AP and oblique view). Images were retrospectively reviewed by a board-certified musculoskeletal radiologist (M.P. with 11 years of experience) and senior musculoskeletal radiologist (I.S.S. with 15 years of experience) and were blinded to clinical and laboratory data.

Foot and ankle radiographs were analyzed for signs of joint inflammation including osteoporosis, soft tissue swelling, joint space narrowing (JSN), erosions and subchondral cysts, ankylosis, malalignment, and growth abnormalities. Non-inflammatory findings were also analyzed including: hallux valgus deformity, pes planus, anatomical variants, benign lesions (unfused ossification center, tarsal coalition, bone island, fibrous cortical defect, osteochondral defect) and osteochondroses. Then the cohort was divided into two groups: patients with confirmed JIA and control group of non-JIA children with foot and ankle arthralgia without final diagnosis of inflammatory connective tissue disease. The final diagnosis of JIA was based on clinical history and symptoms, with differential diagnosis of other than rheumatic background and supported by imaging including radiography and US. Radiographic findings in both groups were compared.

In 211 patients with negative radiographs additional US of foot and ankle was performed. A MRI was only done in six patients. Laboratory data for all patients were collected, including C-reactive protein (CRP; cut-off value 10 mg/l) and erythrocyte sedimentation rate level (ESR; cut-off value 15 mm/h), the presence of antinuclear antibodies (ANA; titre higher or equal to 1:160), anticyclic citrullinated peptide antibodies (anti-CPP; cut-off value 17 IU/ml), rheumatoid factor (RF; cut-off value 34 IU/ml), and human leukocyte antigen (HLA) B-27 antigen. All patients were also tested for Borrelia burgdorferi, Yersinia enterocolitica, hepatitis type C, Tuberculosis infection as well as analyzing Vitamin D levels.

This study was approved by the local institutional review board in accordance with the Helsinki Declaration and amendments (number KBT-3/3/2018).

## Statistical analysis

The results are presented as percentage for categorical variables, mean with 1 standard deviation for normally distributed continuous variables, or median (range) for non-normally distributed continuous variables as tested by the Shapiro–Wilk test. A p value of less than 0.05 was considered significant. The differences between analyzed groups were compared by the U Mann–Whitney test for continuous variables. The prevalence of variables was assessed by the Chi-square test, the Chi-square test with Yates correction, Fisher Freeman Halton or Fisher exact test. Statistical analysis was performed using CytelStudio version 10.0, created January 16, 2013 (CytelStudio Software Corporation, Cambridge, Massachusetts, United States), and Statistica version 10, 2011 (Stat Soft, Inc., Tulsa, Oklahoma, United States).

## Results

From a total number of 372 included patients, 165 patients had final diagnosis of JIA and in 207 children arthralgia without diagnosis of connective tissue disease was recognized. Table 1 lists the characteristics with no significant differences (p > 0.05) found between both groups.

**Table 1:** Subject characteristics.


	JUVENILE IDIOPATHIC ARTHRITIS	CONTROL GROUP, PATIENTS WITH FOOT AND ANKLE ARTHRALGIA	p VALUE

Total number	165	207	–

Gender (% of subgroup)	Male: 64 (38.8%)	Male: 70 (33.8%)	0.321

	Female: 101 (61.2%)	Female: 137 (66.2%)	

Average age (years)	10.70	10.73	0.978

[STDEV]	[4.20]	[4.19]	

Median (min-max)	12 (1–18)	11 (2–18)	


**Figure 1:**
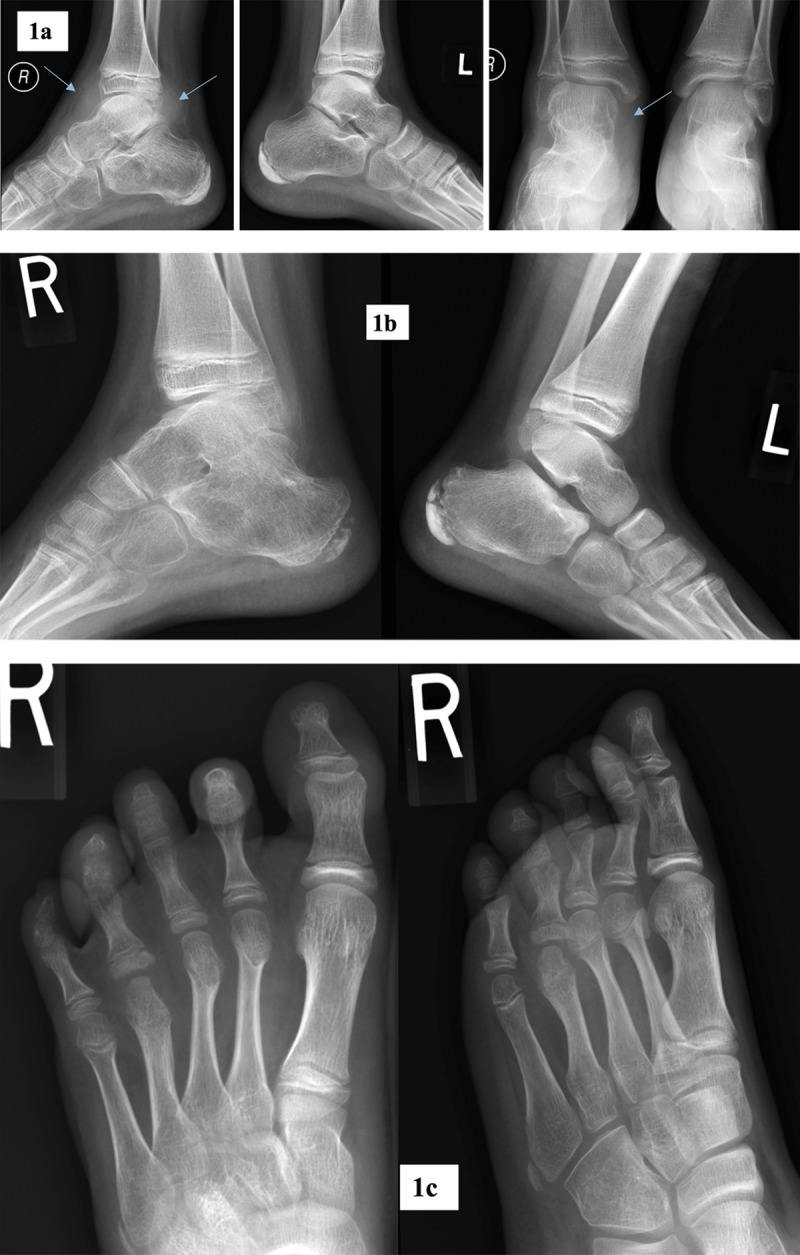
Radiographic abnormalities of foot and ankle. **a.** Antero-posterior and lateral radiographs of both ankle joints in a 12-year-old male patient with JIA. Marked soft tissue swelling at the level of right ankle joint (arrows). **b.** Lateral radiographs of ankle joints in a 9-years-old male with JIA. Marked osteoporosis of right talar and calcaneal bones with ankylosis in subtalar joint. Talonavicular joint space narrowing with subchondral sclerosis. Normal left ankle joint. **c.** Antero-posterior and oblique radiographs of the right foot in a 9-year-old female with JIA. Erosions and subchondral cysts in fourth MTP and PIP joints, periarticular osteoporosis, soft tissue swelling at the level of MTP2 joint.

In the JIA group radiographic abnormalities were found in 99/165 patients (60%). In the control group of non-JIA patients with foot and ankle arthralgia abnormal findings were reported in 62/207 patients (29.95%). Statistically significant correlation between diagnosis of JIA and the presence of abnormalities has been found (p = 0.000). Soft tissue swelling (Figure 1a and Table 2) was reported in 31.51% children with JIA and in only 2.41% patients in control group with statistical significance (p = 0.000).

**Table 2:** Radiographic findings in JIA and non-JIA groups.


1. INFLAMMATORY LESIONS								

	SOFT TISSUE SWELLING	OSTEO-POROSIS	JSN	EROSIONS AND SUBCHONDRAL CYSTS	ANKYLOSIS	MALALIGNMENT	GROWTHABNORMALITIES	PERIOSTEAL BONE FORMATION

**JIA** (165 patients)	52 (31.51%)	24 (14.55%)	10 (6%)	15 (9.09%)	2 (1.21%)	1 (0.6%)	1 (0.6%)	5 (3.03%)

**non-JIA** (207 patients)	5 (2.41%)	2 (0.97%)	0	12 (5.79%)	0	0	0	1 (0.48%)

P	p = 0.000	p = 0.000	p = 0.000	p > 0.05	p > 0.05	p > 0.05	p > 0.05	p > 0.05

**2. NON- INFLAMMATORY LESIONS**								

	**PES PLANUS**	**HALLUX VALGUS**	**ANATOMICAL VARIANTS AND BENIGN LESIONS (UNFUSED OSSIFICATION CENTER, TARSAL COALITION, BONE ISLAND, FIBROUS CORTICAL DEFECT, OSTEOCHONDRAL DEFECT)**				**ASEPTIC NECROSIS**	

**JIA** (165 patients)	34 (20.6%)	11 (6.66%)	6 (3.63%)				4 (2.42%)	

**non-JIA** (207 patients)	40 (19.32%)	20 (9.67%)	10 (4.83%)				3 (1.45%)	

P	p > 0.05	p > 0.05	p > 0.05				p > 0.05	


* JSN joint space narrowing.

Statistically significant correlation was also found between elevated ESR and soft tissue swelling (p < 0.05) but for CRP there was no statistical correlation.

In 34/52 (65.38%) cases soft tissue swelling was the only abnormality in JIA patients and the most frequent finding occurring concomitantly was osteoporosis (11/52; 21.15%).

**Figure 2:**
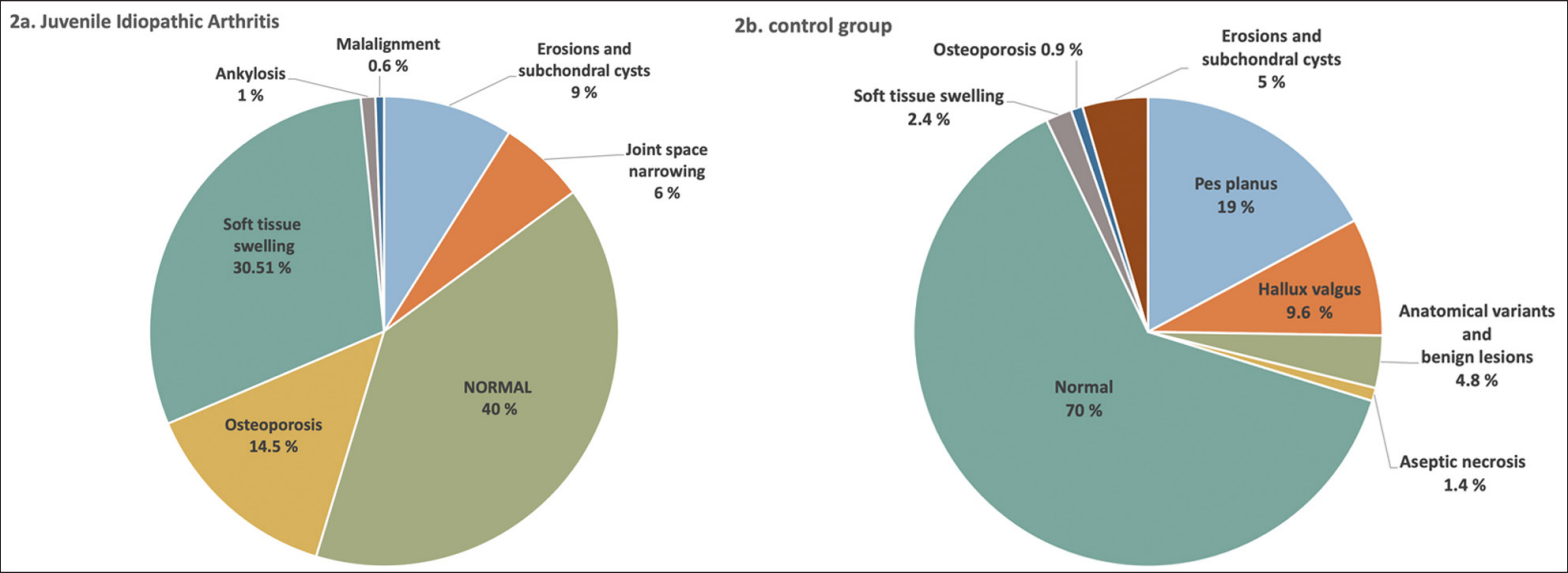
Radiographic findings. **a.** In the JIA group. The diagram covers only inflammatory changes. Non-inflammatory lesions, also detected in JIA group, because of overlapping were not included. **b.** In the non-JIA group.

Osteoporosis was the second most commonly recognized lesion found in JIA group (Figure 1b) with statistically significant correlation (p = 0.000) followed by JSN (Figure 1b and Table 2). Erosions and subchondral cysts were found in both groups (p > 0.05) (Figure 1c and Table 2). All remaining inflammatory lesions, although recognized more commonly in JIA than in non-JIA group, did not meet statistical significance. Among non-inflammatory radiographic findings none met a statistical significance when compared in both groups (Table 2). In control group the vast majority had non-inflammatory findings (Table 2, Figure 2a–b).

The most frequent JIA subtype was oligoarthritis, Table 3 include the frequency of all recognized subtypes. Comparison between JIA subtypes showed that the plain film abnormalities were most frequent in oligoarthritis 50/76 (65.78%). The most commonly detected pathology in oligoarthritis was soft tissue swelling in 27/76 (35.52%) cases followed by osteoporosis in 10/76 (13.15%) subjects, erosions and subchondral cysts in 8/76 (10.52%) cases, periosteal new bone formation in 3/76 (3.94%) cases, malalignment in 1/72 (1.38%) and ankylosis in 1/72 (1.38%) patients.

**Table 3:** JIA recognized subtypes.


JIA SUBTYPES	NUMBER (PERCENTAGE)

Oligoarthritis	76 (46%)

Polyarthritis:	57 (34.54%)

RF- negative	54

RF-positive	3

ERA (entesitis-related)	2 (1.21%)

Undifferentiated form	25 (15.15%)

Systemic form	5 (3.03%)


Yersinia enterocolitica infection was diagnosed in 14/372 (3.76%) subjects. Borrelia burgdorferi infection diagnosed with ELISA test confirmed with Western blot test was found in 6/372 (1.61%) cases, of which only one was diagnosed with JIA. Positive test for tuberculosis was found in 9/372 (2.41%) cases. There were no cases of diagnosed hepatitis C infection. Vitamin D deficiency was found in 102/372 (27.41%) subjects, including 63 children in the control group and 39 with diagnosed JIA.

**Figure 3:**
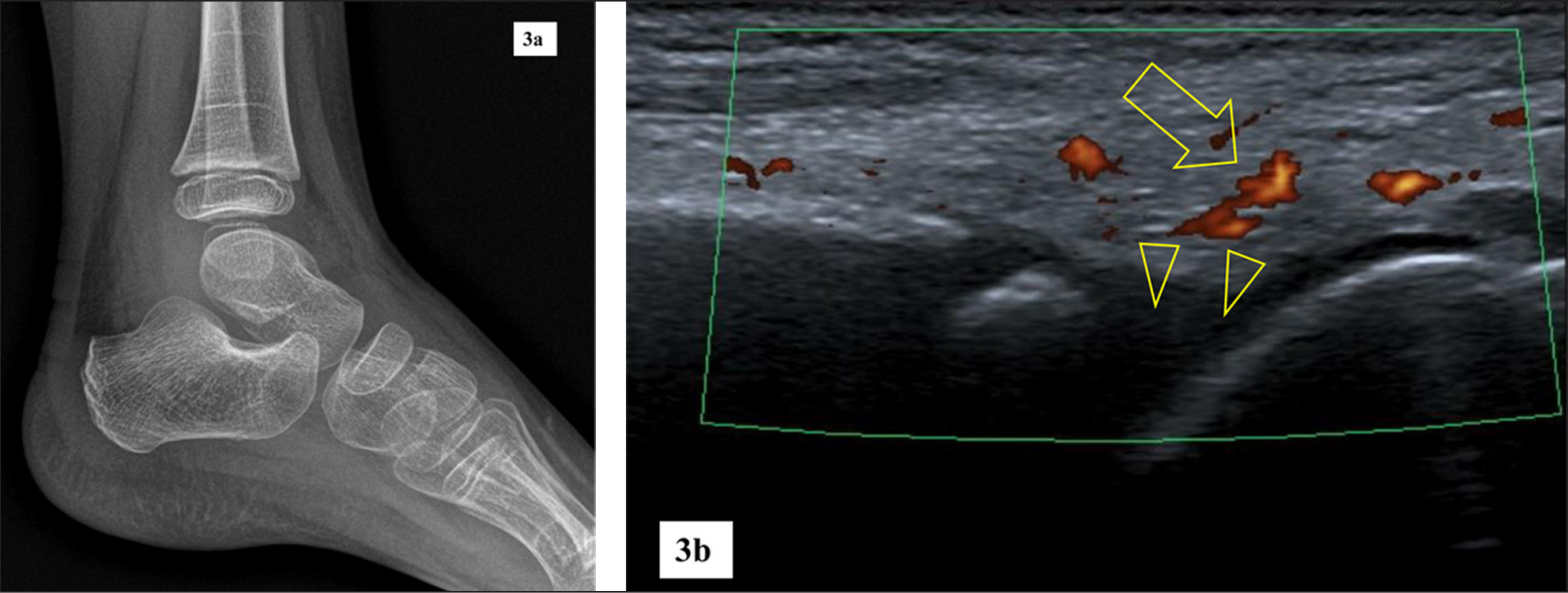
5-year-old girl with confirmed JIA. **a.** Lateral radiograph of a left ankle is unremarkable. **b.** Sagittal ultrasound of the left ankle shows hypervascular synovium on Power Doppler mode (arrow) and small tibiotalar effusion (arrowheads).

From 211 patients with negative plain films subsequent ankle and/or foot US imaging has been performed in 191 cases and showed abnormalities in 101 cases (63 non-JIA and 38 JIA cases) (Figure 3a–b). Findings included joint effusion, (teno)synovitis, subcutaneous oedema, ganglion cysts and bursitis. Tarsal bone marrow edema was the predominant MRI finding.

Laboratory results are listed in Table 4, without significant correlation with final diagnosis.

**Table 4:** Laboratory results.


	JIA NUMBER OF PATIENTS		NON-JIA NUMBER OF PATIENTS	
	
	NEGATIVE	POSITIVE	NEGATIVE	POSITIVE

**Antinuclear antibodies (ANA)**	49	116	72	135

**Anticyclic citrullinated peptide antibodies (anti-CPP)**	161	4	207	0

**Rheumatoid factor (RF)**	162	3	206	1

**Human leukocyte antigen (HLA) B-27 antigen**	126	39	189	18

**Erythrocyte sedimentation rate level (ESR)**	Not elevated in 81	Elevated in 84	Not elevated in 161	Elevated in 46

**C-reactive protein (CRP)**	Not elevated 120	Elevated in 45	Not elevated in 196	Elevated in 11


## Discussion

The dominant radiographic finding at the level of foot and ankle in patients with newly recognized JIA is soft tissue swelling. Together with osteoporosis and JSN those abnormalities met significant correlation with final diagnosis of JIA when compared to control group. Our results are in line with previous studies evaluating the most common radiographic findings in JIA [10].

Nowadays the main focus in patient care is to prevent from irreversible bone damage, which in past was more commonly recognized on radiographs [7]. Radiography remains valuable not only in initial imaging but also as a tool for detecting radiographic progression [9]. Prospective study of early JIA showed that radiographic abnormalities not only tend to appear early but may also progress after three year-follow up in up to 38% [3].

On radiographs, soft tissue swelling is seen as increase of joint outlines and increased radiodensity of tissues. Although the sign itself is not helpful in differential diagnosis of inflammatory, infectious and other causes [13,14], it is the most common lesion in JIA [7] that for a long time may be the only abnormality.

Soft tissue swelling may be due to joint effusion and synovitis. The overall drawback of radiography is that it is far less sensitive in detection of small amount of joint fluid. Radiographically detectable ankle effusion requires and amount of 5 ml, whereas MR and ultrasound may reveal the amount of 1 ml and 2 ml respectively [15].

Furthermore, synovitis in the lower extremity often appears in subclinical form, especially at the level of MTP joints and frequently remains underestimated on clinical exam and radiography [11]. In these scenarios, subsequent US or MRI may be mandatory to detect inflammation in radiographically negative JIA patients allowing early treatment resulting in a better outcome [12].

The second most common lesion was osteoporosis. If present, it is a worrisome sign requiring further investigation. Compared to radiography, ultrasound is of no value for detection of osteoporosis. Osteoporosis in children is predominantly secondary with a few underlying primary conditions including genetic disorders. Mostly it is related to systemic inflammatory diseases, endocrine disorders or corticosteroid use [16]. Malabsorption with Vitamin D deficiency may also be one of predisposing factors, although in this study no significant statistical correlation with radiographic abnormalities was found.

JSN was the third most commonly diagnosed lesion, found solely in JIA patients. According to American College of Rheumatology JSN and erosions are features of poor prognosis [17]. Compared to radiography, ultrasound is very limited to evaluate JSN.

Importantly, in pediatric population due to the relative thick articular cartilage, detection of early erosive lesions on radiographs is limited [7].

Interestingly, cysts and erosions were seen in this study in both groups. Previous studies showed that erosions may be present in up to 11% of healthy controls [18], thus their diagnosis must be made in conjunction with all clinical data.

Except elevated ESR none of laboratory blood tests showed statistical correlation with radiographic abnormalities. Elevated inflammatory markers including ESR and CRP are usually present in systemic or polyarticular form of the disease, but in oligoarthritis, which was the dominant type in the study, these parameters mostly are within reference range. Nevertheless, laboratory tests play role in differential diagnosis, classification of the type of arthritis and prognosis [5,17].

Although radiographic abnormalities were found statistically significant for JIA when compared to control group, 40% of patients with newly recognized disease had normal plain films. This can be related to radiographically occult inflammatory soft tissue changes [19].

Patient’s complaints in our control group may be explained by some abnormalities not related to inflammatory disease, although clinical appearance may suggest JIA. The recognition of non-inflammatory bone changes in non-JIA group was crucial in the final alternative diagnosis other than JIA [20].

The strength of this study is the inclusion of a large number of patients hospitalized in tertiary referral center for rheumatologic diseases with experienced team of clinicians and radiologists. Each patient underwent a full set of laboratory tests excluding infectious background, evaluating inflammatory parameters with profound clinical assessment.

The main limitation of this study is the use of a control group which included children suffering from arthralgia related to various causes. Due to ethic reasons in the use of ionizing radiation in pediatric population it was difficult to enroll healthy volunteers.

Another limitation is that systematic comparison with US and/or MRI on a fixed date was not available in our patient cohort.

## Conclusion

Soft tissue swelling, osteoporosis and JSN are significantly more common in JIA patients as compared with patients without inflammatory connective tissue disease. Conventional radiography supports the diagnosis of JIA and is still a very important part of initial diagnostic algorithm in foot and ankle arthralgia of unknown etiology with indisputable role in primary differential diagnosis and follow up. However, as radiography is normal in 40% of JIA, in any case of clinical suspicion of JIA affecting the ankle/foot with a negative radiography, the examination should be supplemented with US and/or MRI to detect early changes in order to allow prompt treatment resulting is a favorable prognosis.

## References

[1] Esbjornsson AC, Aalto K, Brostrom EW, et al. Ankle arthritis predicts polyarticular disease course and unfavourable outcome in children with juvenile idiopathic arthritis. Clin Exp Rheumatol. 2015; 33(5): 751–7. 26213158

[2] Flato B, Lien G, Smerdel-Ramoya A, Vinje O. Juvenile psoriatic arthritis: Long-term outcome and differentiation from other subtypes of juvenile idiopathic arthritis. J Rheumatol. 2009; 36(3): 642–50. DOI: 10.3899/jrheum.080543 19208605

[3] Selvaag AM, Flato B, Dale K, et al. Radiographic and clinical outcome in early juvenile rheumatoid arthritis and juvenile spondyloarthropathy: A 3-year prospective study. J Rheumatol. 2006; 33(7): 1382–91. 16758503

[4] Sudol-Szopinska I, Jans L, Jurik AG, et al. Imaging features of the juvenile inflammatory arthropathies. Semin Musculoskelet Radiol. 2018; 22(2): 147–65. DOI: 10.1055/s-0038-1639468 29672804

[5] Crayne CB, Beukelman T. Juvenile idiopathic arthritis: Oligoarthritis and polyarthritis. Pediatr Clin North Am. 2018; 65(4): 657–74. DOI: 10.1016/j.pcl.2018.03.005 30031492

[6] Bugni Miotto e Silva V, de Freitas Tavares da Silva C, de Aguiar Vilela Mitraud S, et al. Do patients with juvenile idiopathic arthritis in remission exhibit active synovitis on joint ultrasound? Rheumatol Int. 2014; 34(7): 937–45. DOI: 10.1007/s00296-013-2909-7 24318644

[7] Sheybani EF, Khanna G, White AJ, Demertzis JL. Imaging of juvenile idiopathic arthritis: A multimodality approach. Radiographics. 2013; 33(5): 1253–73. DOI: 10.1148/rg.335125178 24025923

[8] Bechtold S, Simon D. Growth abnormalities in children and adolescents with juvenile idiopathic arthritis. Rheumatol Int. 2014; 34(11): 1483–8. DOI: 10.1007/s00296-014-3022-2 24760485

[9] Malattia C, Rinaldi M, Martini A. The role of imaging in juvenile idiopathic arthritis. Expert Rev Clin Immunol. 2018; 14(8): 681–94. DOI: 10.1080/1744666X.2018.1496019 29972659

[10] Breton S, Jousse-Joulin S, Finel E, et al. Imaging approaches for evaluating peripheral joint abnormalities in juvenile idiopathic arthritis. Semin Arthritis Rheum. 2012; 41(5): 698–711. DOI: 10.1016/j.semarthrit.2011.08.004 22035628

[11] Breton S, Jousse-Joulin S, Cangemi C, et al. Comparison of clinical and ultrasonographic evaluations for peripheral synovitis in juvenile idiopathic arthritis. Semin Arthritis Rheum. 2011; 41(2): 272–8. DOI: 10.1016/j.semarthrit.2010.12.005 21377713

[12] Hendry GJ, Gardner-Medwin J, Steultjens MP, et al. Frequent discordance between clinical and musculoskeletal ultrasound examinations of foot disease in juvenile idiopathic arthritis. Arthritis Care Res (Hoboken). 2012; 64(3): 441–7. DOI: 10.1002/acr.20655 21972178

[13] Yi A, Kennedy C, Chia B, Kennedy SA. Radiographic soft tissue thickness differentiating pyogenic flexor tenosynovitis from other finger infections. J Hand Surg Am. 2019; 44(5): 394–9. DOI: 10.1016/j.jhsa.2019.01.013 30797654

[14] Ording Muller LS, Humphries P, Rosendahl K. The joints in juvenile idiopathic arthritis. Insights Imaging. 2015; 6(3): 275–84. DOI: 10.1007/s13244-015-0406-0 25903287 PMC4444796

[15] Jacobson JA, Andresen R, Jaovisidha S, et al. Detection of ankle effusions: Comparison study in cadavers using radiography, sonography, and MR imaging. AJR Am J Roentgenol. 1998; 170(5): 1231–8. DOI: 10.2214/ajr.170.5.9574591 9574591

[16] Uziel Y, Zifman E, Hashkes PJ. Osteoporosis in children: Pediatric and pediatric rheumatology perspective: A review. Pediatr Rheumatol Online J. 2009; 7: 16. DOI: 10.1186/1546-0096-7-16 19835571 PMC2768686

[17] Beukelman T, Patkar NM, Saag KG, et al. 2011 American College of Rheumatology recommendations for the treatment of juvenile idiopathic arthritis: Initiation and safety monitoring of therapeutic agents for the treatment of arthritis and systemic features. Arthritis Care Res (Hoboken). 2011; 63(4): 465–82. DOI: 10.1002/acr.20460 21452260 PMC3222233

[18] Tamas MM, Filippucci E, Becciolini A, et al. Bone erosions in rheumatoid arthritis: Ultrasound findings in the early stage of the disease. Rheumatology (Oxford). 2014; 53(6): 1100–7. DOI: 10.1093/rheumatology/ket484 24501246

[19] Damasio MB, Malattia C, Martini A, Toma P. Synovial and inflammatory diseases in childhood: Role of new imaging modalities in the assessment of patients with juvenile idiopathic arthritis. Pediatr Radiol. 2010; 40(6): 985–98. DOI: 10.1007/s00247-010-1612-z 20432018

[20] Houghton KM. Review for the generalist: evaluation of pediatric foot and ankle pain. Pediatr Rheumatol Online J. 2008; 6: 6. DOI: 10.1186/1546-0096-6-6 18400098 PMC2323000

